# Growth Parameters and Survivability of *Saccharomyces boulardii* for Probiotic Alcoholic Beverages Development

**DOI:** 10.3389/fmicb.2019.02092

**Published:** 2019-09-10

**Authors:** Breno Pereira de Paula, Davy William Hidalgo Chávez, Wilson José Fernandes Lemos Junior, André Fioravante Guerra, Mariana Ferreira Dutra Corrêa, Karen Signori Pereira, Maria Alice Zarur Coelho

**Affiliations:** ^1^Coordenadoria do Curso de Engenharia de Alimentos, Centro Federal de Educação Tecnológica Celso Suckow da Fonseca, Valença, Brazil; ^2^Programa de Pós-Graduação em Ciência de Alimentos, Universidade Federal do Rio de Janeiro, Rio de Janeiro, Brazil; ^3^Departamento de Tecnologia de Alimentos, Universidade Federal Rural do Rio de Janeiro, Seropédica, Brazil; ^4^Department of Biotecnology, University of Verona, Verona, Italy; ^5^Escola de Química, Universidade Federal do Rio de Janeiro, Rio de Janeiro, Brazil

**Keywords:** functional beverage, alcoholic resistance, yeast, gut, dysbiosis

## Abstract

The aim of this research was to optimize the growth parameters (pH, ethanol tolerance, initial cell concentration and temperature) for *Saccharomyces boulardii* and its tolerance to *in vitro* gastrointestinal conditions for probiotic alcoholic beverage development. Placket-Burman screening was used to select only statistically significant variables, and the polynomial mathematical model for yeast growth was obtained by central composite rotatable design. Confirmation experiments to determine the kinetic parameters for yeast growth were carried out by controlling the temperature and pH. Soon after, the survivability of yeast was tested under *in vitro* conditions mimicking the human upper gastrointestinal transit. *S. boulardii* had suitable resistance to alcohol and gastrointestinal conditions for probiotic alcoholic beverage development.

## Introduction

Eating habits have changed over the decades in pursuit of a healthier diet. Therefore, food and drink are being consumed with the purpose of disease prevention or treatment instead of only basic nutrition. Probiotics are live microorganisms that, when administered in adequate amounts, confer a health benefit on the host (Joint FAO/WHO Working Group, 2002). Properly controlled studies on probiotics microbial to confer benefits to health have been included to the probiotic definitions. Additionally, new commensals and consortia comprising defined strains from human samples, with adequate evidence of safety and efficacy, are also “probiotics” (Hill et al., 2014). Probiotic food matrices should ensure the survivability of probiotic microorganisms throughout the gastrointestinal tract (Spigno et al., 2015). The majority of probiotic strains are intended for gut health improvement (Floch, 2017). However, probiotic strains must resist gastrointestinal conditions to benefit gut health.

Lactic acid bacteria are the main strains used as probiotics, but some *Saccharomyces* sp., *Escherichia coli* and *Bacillus* sp. have also been employed (Fijan, 2014). *Saccharomyces* sp. is a yeast, belonging to the kingdom Fungi, and has suitable features for brewing, such as the ability to metabolize high sugar concentrations in a large range of pH values, nitrogen levels, and temperatures (Lemos Junior et al., 2017). *Saccharomyces boulardii* is classified as a subtype of *Saccharomyces cerevisiae* and was first isolated from mangosteen and lychee by Henri Boulardii in 1923 (Altmann, 2018). *S. boulardii* is unique probiotic and biotherapeutic yeast, known to survive in gastric acidity and it is not adversely affected or inhibited by antibiotics or does not alter or adversely affect the normal micro biota (Tomičić et al., 2016). Its consumption provides several benefits for human health, such as travelers’ diarrhea, irritable bowel syndrome (IBS), colitis and related malaise, such as inflammatory bowel, Crohn’s disease, acute gastroenteritis, chronic diarrhea in HIV-infected caused by *Clostridium difficile*, *Vibrio cholerae*, and other pathogenic enterobacteria (Szajewska and Kołodziej, 2015; Bagherpour et al., 2018; Terciolo et al., 2019).

Regarding to methodologies, the development, production or optimization of new products are not a simple task due to the high cost of process and reagents and the increments of requirements of regulatory agencies. In this sense, the design of experiments (DOE) and response surface methodology (RSM) are important issues because its takes less time, effort, and resources as well as facilitates the collection of a large amount of information with minimizing the number of experiments (Candioti et al., 2014). RSM has been demonstrated to be efficient for developing, improving, and optimizing processes, and it has been broadly used in analytical applications, industrial world, and bioprocesses (Adinarayana et al., 2003; Kristo et al., 2003; Lee and Gilmore, 2005; Zhi et al., 2005; Myers et al., 2016).

On the other hand, alcoholic fermented beverages are consumed extensively worldwide, mainly for the welfare (Silva et al., 2016). Although a lot of information about an alcoholic beverage, researchers regarding probiotic alcoholic beverages and its optimization by applying RSM are needing. Thus, development probiotic alcoholic beverages present new challenge and possibilities for the delivery of probiotic yeast and add some functional features to alcoholic beverage consumption.

For the reason above mentioned, the aim of this research was to applying RSM to optimize the growth parameters for *S. boulardii* (pH, alcohol content, initial cell concentration, and temperature) in synthetic must and its tolerance under conditions that mimic *in vivo* human upper gastrointestinal transit, thereby facilitating probiotic alcoholic beverage development.

## Materials and Methods

### Microbial Isolation and Standardization of Working Inoculum

*Saccharomyces boulardii* – 17 (Floratil^®^ 200, Merck, France) was obtained by streaking on Sabouraud agar media (HiMedia, Mumbai, India) followed by incubation at 25°C for 48 h. A single colony was picked with a sterile loop and transferred to a tube containing yeast-extract-peptone-dextrose (YPD) broth. After incubation overnight at 25°C, the working inoculum was obtained in YPD at the same temperature under gentle shaking (160 min^–1^). After growth, the cells were harvested by centrifugation at 6000 × *g* for 5 min. The liquid fraction was discarded, and the remaining cell pellet was washed twice with phosphate buffer pH 7.2 and resuspended in the same buffer up to an optical density at 620 nm (OD_620__nm_) measurement corresponding to ca. 10^8^ cfu mL^–1^.

### Determination of the Cellular Concentration

Cell concentration determinations performed during the culture of *S. boulardii* were expressed as dry weight (X_dw_) and viable cells (X_vc_).

Dry weight (Log_10_ g dry weight L^–1^) were determined from centrifuged samples by discarding the supernatant and resuspending the pellet in 100 mL of distilled water. Subsequently, the cell suspension was diluted 1:100 to quantify the optical density at 620 nm (OD_620__nm_) in a spectrophotometer (Biospectro model Spectrum SP-2000UV, Brazil) within the linearity range (0.050 to 0.500). The OD_620__nm_ values were related to the dry weight of the cell suspensions filtered on cellulose acetate membranes with 0.45 μm pore diameter and dehydrated in a chamber with infrared radiation to construct a calibration curve and obtain the conversion factor of OD_620__nm_ in Dry weight.

Viable cells (Log_10_ viable cells mL^–1^) was determined by mixing equal parts of the appropriately diluted cell suspension with methylene blue solution (0.1% m v^–1^). The solution was observed in a Neubauer chamber using an optical microscope [Biofocus (R), United States].

### Substrate Consumption and Product Generation Measurement

*Saccharomyces boulardii* metabolic sugar consumption (glucose) and product formation (glycerol, ethanol, and acetic acid) were analyzed using a high-performance liquid chromatography system equipped with an infrared detector, binary pump, control module, and LabSolution chromatography software (Shimadzu^®^, Japan). The chromatography column used was an Aminex^®^ HPX-87H 300 mm × 7.8 mm (Bio-Rad Laboratories Ltd., United States). The mobile phase was prepared in 5 mM H_2_SO_4_ at a flow rate of 0.8 mL min^–1^. The injection volume was set to 20 μL, and the temperature was set to 60°C.

### pH Measurement

A benchtop pH meter (MPA-201, Tecnopon, Brazil) with a digital microprocessor equipped with automatic temperature compensation, calibrated with pH 4.0 buffer (citric acid, sodium hydroxide, and hydrogen chloride, Merck^®^, France) and pH 7.0 buffer (potassium dihydrogen phosphate and disodium hydrogen phthalate, Merck^®^, France), was used for pH measurements.

### Optimizing *Saccharomyces boulardii* Growth Conditions

*Saccharomyces boulardii* growth optimization was carried out by determining independent variables with significant influence on growth, in accordance with Plackett and Burman matrix (Plackett and Burman, 1946); optimization by central composite rotatable design (CCRD); and polynomial mathematical model validation. Plackett and Burman design was made with a $2_{\text{IV}}^{4-1}$ fractional factorial experiment (four factors), each of them with two levels, and three central points be able to check the curvature, the mathematical model for Plackett and Burman is in Eq. 1.

(1) $$Y=\beta_{0}+\sum_{i=1}^{k}\beta_{i}\,X_{i}+\sum_{i=1}^{k}\beta_{i\,i}\,X_{i}^{2}$$

Where Y is predicted response; i and ii are linear and quadratic coefficients, respectively; β are regression coefficient and *k* is the number of factors.

One possible design to evaluated the four independent variables (alcohol content – AC, potential of hydrogen – pH, initial cell concentration – X_0_, and temperature – T) could be the 2^k^ full factorial (*k* = 4). In this case, the total number of treatments will be 16 and, considering three repetitions, it will totalize 48 runs. Another disadvantage of full 2^k^ factorial design would be that it only have two levels, and it will be impossible to detect the possible curvature effect. A 3^k^ full factorial (*k* = 4) is able to evaluate the curvature effect but it would have 81 treatments totalizing 243 runs with 3 repetitions. On the other hand, a Plackett and Burman design with central points for four variables only need 11 runs with the advantage in detecting the possible curvature effect. Thus, four independent variables (alcohol content – AC, potential of hydrogen – pH, initial cell concentration – X_0_, and temperature – T) were evaluated for screening performance at three different levels. Eight factorial point combinations (±1) and three central points, for a total of 11 trials (Table 1), were generated. Miniaturized tests (10 mL, screw-cap tubes) were performed in accordance with the experimental design in YPD. Biomass formation (X_dw_), measured by OD_620__nm_ every 24 h, was the dependent variable.

**TABLE 1:** Eleven trials in Plackett and Burman (PB 8) experimental design for *Saccharomyces boulardii* growth.

**Test**	**AC**	**pH**	**X_0_**	**T**	**X_dw_ ± SD**
1	+1 (11.30)	−1 (3.30)	−1 (1 × 10^2^)	+1 (21.3)	−1.85 ± 0.00
2	+1 (11.30)	+1 (5.80)	−1 (1 × 10^2^)	−1 (13.8)	−1.59 ± 0.02
3	+1 (11.30)	+1 (5.80)	+1 (1 × 10^5^)	−1 (13.8)	−1.54 ± 0.01
4	−1 (3.80)	+1 (5.80)	+1 (1 × 10^5^)	+1 (21.3)	−0.54 ± 0.01
5	−1 (3.80)	−1 (3.30)	+1 (1 × 10^5^)	+1 (21.3)	−2.03 ± 0.02
6	−1 (3.80)	+1 (5.80)	−1 (1 × 10^2^)	+1 (21.3)	−0.54 ± 0.00
7	+1 (11.30)	−1 (3.30)	+1 (1 × 10^5^)	−1 (13.8)	−2.44 ± 0.06
8	−1 (3.80)	−1 (3.30)	−1 (1 × 10^2^)	−1 (13.8)	−2.32 ± 0.06
9	0 (7.55)	0 (4.55)	0 (5 × 10^4^)	0 (17.6)	−0.61 ± 0.01
10	0 (7.55)	0 (4.55)	0 (5 × 10^4^)	0 (17.6)	−0.57 ± 0.03
11	0 (7.55)	0 (4.55)	0 (5 × 10^4^)	0 (17.6)	−0.64 ± 0.01

*AC, alcohol content (°GL); pH, potential of hydrogen; X_0_, initial cell concentration (cells mL^–1^); T, incubation temperature (°C); X_*dw*_, biomass formation (Log_10_ g of dry weight L^–1^); SD, standard deviation (*n* = 3). Real values are shown in parentheses.*

From the screening results, central composite rotatable design was performed using the independent variables alcohol content, pH, and temperature. Cellular concentration in dry weight and viable cells were used as dependent variables. Five levels by dependent variable including eight factorial points (±1), six axial points (±α), and five central points to estimate the pure error were evaluated, for a total of 19 trials (Table 2). The mathematical model for CCRD is presenting in Eq. 2.

**TABLE 2:** Central composite rotatable design (CCRD 2^3^) for *S. boulardii* growth.

**Teste**	**AC**	**pH**	**T**	**Biomass formation**			**Viable cells**		
				**X_dw_ ± SD**	**PV**	**RD**	**X_vc_ ± SD**	**PV**	**RD**
1	−1 (3.0)	−1 (5.00)	−1 (18.5)	−0.60 ± 0.01	−0.53	–12.20	8.16 ± 0.02	8.18	0.24
2	−1 (3.0)	−1 (5.00)	+1 (23.5)	−0.36 ± 0.00	−0.33	–9.01	8.56 ± 0.01	8.51	–0.61
3	−1 (3.0)	+1 (6.00)	−1 (18.5)	−0.57 ± 0.02	−0.53	–8.01	8.45 ± 0.02	8.49	0.46
4	−1 (3.0)	+1 (6.00)	+1 (23.5)	−0.30 ± 0.00	−0.24	–26.45	8.55 ± 0.02	8.61	0.69
5	+1 (11.8)	−1 (5.00)	−1 (18.5)	−2.19 ± 0.04	−1.92	–14.05	6.61 ± 0.04	6.83	3.21
6	+1 (11.8)	−1 (5.00)	+1 (23.5)	−2.36 ± 0.03	−2.06	–14.38	6.56 ± 0.04	6.80	3.47
7	+1 (11.8)	+1 (6.00)	−1 (18.5)	−2.13 ± 0.02	−1.82	–17.10	6.71 ± 0.06	7.04	4.63
8	+1 (11.8)	+1 (6.00)	+1 (23.5)	−2.15 ± 0.10	−1.88	–14.60	6.54 ± 0.06	6.80	3.76
9	−1.68 (0.0)	0 (5.50)	0 (21.0)	−0.31 ± 0.00	−0.29	–8.38	8.59 ± 0.05	8.67	0.96
10	+1.68 (14.8)	0 (5.50)	0 (21.0)	−2.31 ± 0.14	−2.83	18.35	6.48 ± 0.01	6.01	–7.80
11	0 (7.4)	−1.68 (4.66)	0 (21.0)	−0.75 ± 0.00	−0.99	24.57	7.81 ± 0.02	7.68	–1.74
12	0 (7.4)	+1.68 (6.34)	0 (21.0)	−0.57 ± 0.01	−0.83	31.43	8.20 ± 0.03	7.94	–3.33
13	0 (7.4)	0 (5.50)	−1.68 (16.8)	−0.74 ± 0.01	−0.99	25.46	8.08 ± 0.01	7.86	–2.82
14	0 (7.4)	0 (5.50)	+1.68 (25.2)	−0.62 ± 0.00	−0.87	28.64	8.11 ± 0.00	7.93	–2.25
15	0 (7.4)	0 (5.50)	0 (21.0)	−0.66 ± 0.00	−0.65	–1.77	8.10 ± 0.01	8.10	–0.03
16	0 (7.4)	0 (5.50)	0 (21.0)	−0.66 ± 0.00	−0.65	–1.77	8.08 ± 0.00	8.10	0.22
17	0 (7.4)	0 (5.50)	0 (21.0)	−0.66 ± 0.00	−0.65	–1.77	8.09 ± 0.01	8.10	0.10
18	0 (7.4)	0 (5.50)	0 (21.0)	−0.66 ± 0.00	−0.65	–1.77	8.06 ± 0.02	8.10	0.47
19	0 (7.4)	0 (5.50)	0 (21.0)	−0.66 ± 0.00	−0.65	–1.77	8.09 ± 0.03	8.10	0.10

*AC, alcohol content (°GL); pH, potential of hydrogen; T, incubation temperature (°C); X_*dw*_, biomass formation (Log_10_ g of dry weight L^–1^); X_*vc*_, cell concentration (Log_10_ viable cells mL^–1^); SD, standard deviation (*n* = 3); PV, predicted value from polynomial mathematical model; RD, relative deviation. Real values are shown in parentheses.*

(2) $$Y=\beta_{0}+\sum_{i=1}^{k}\beta_{i}\,X_{i}+\sum_{i=1}^{k}\beta_{i\,i}\,X_{i}^{2}+\sum_{i=1}^{k}\sum_{j=1}^{k}\beta_{i\,j}\,X_{i}\,X_{j}$$

Where Y is predicted response; i, ii, and ij are linear, quadratic, and interaction coefficients, respectively; β are regression coefficient; and k is the number of factors.

Additionally, desirability functions was performed to optimization of simultaneous multiple responses. According to Myers et al. (2016), the first approach is to convert each response (*Y*_i_) into an individual desirability (*d*_i_) varying from 0 to 1, and then obtain the overall desirability (D) applying the geometric median according Eq. 3.

(3) $$D=\sqrt[{\sum_{m}}]{d_{1}^{w\,1}.d_{2}^{w\,2}\,\ldots \,d_{m}^{w_{m}}}$$

Were *d*_i_ is the individual desirability, and *m* are the number of responses used to carried out the overall desirability (D) and *w* is the weight attributed for each response.

Each desirability is calculating according the objective for each variable, thus it could target for the response to be at minimum (Eq. 4).

(4) $$d=\left\{ \begin{array}{ccc} 0 & , & y<L\\ {\left(\frac{y-L}{T-L}\right)}^{r} & , & L\leq y\leq T\\ 1 & , & y>T \end{array}\right.$$

Where *r* is a weight used to determine the scale of desirability, if *r* = 1, the desirability function is linear, if *r* > 1 places more emphasis on being close to the target value, and choosing 0 ≤ *r* ≤ 1 makes this less important. *y* is the response value, *L* is the minimum acceptable response, and *T* is the maximum acceptable response (Derrien et al., 2017).

A bioreactor (SL-135, Solab, Brazil) with the working volume filled (5 L) with YPD containing added alcohol (5.0°GL) and the pH adjusted to 5.5 was incubated at 24°C to confirm the adequacy of the developed polynomial mathematical model to maximize yeast growth.

### Determination of Kinetic Parameters

A polynomial mathematical model validation assay was developed to determine the kinetic parameters (generation time – t_g_, specific growth rate – μ_X_, rate of substrate utilization – μ_S_, and conversion factor of substrate into biomass – Y_X/S_) for *S. boulardii* growth (X) and substrate consumption (S), in accordance with Eqs. 5–8.

(5) $$t_{g}=\frac{L\,n\,2}{\mu_{m}}$$

(6) $$\mu_{m}=\frac{1}{X}\,\frac{\mathrm{𝑑𝑋}}{\mathrm{𝑑𝑡}}=\frac{\mathrm{𝑙𝑛}\,X-\mathrm{𝑙𝑛}\,X_{0}}{t-t_{0}}$$

(7) $$Y_{X\,S^{-1}}=-\frac{\mathrm{dX}}{\mathrm{dS}}=-\frac{X-X_{0}}{S-S_{0}}$$

(8) $$\mu_{S}=-\frac{1}{X}\,\frac{\mathrm{dS}}{\mathrm{dt}}=\frac{\mu_{m}}{Y_{X\,S^{-1}}}$$

Here, t_g_ – generation time, from exponential growth phase (h), μ_m_ – specific speed for maximum growth, from log phase (h^–1^), X_0_ – initial concentration of viable cells (cells L^–1^), X – concentration of viable cells (cells L^–1^), S_0_ – initial concentration of glucose (g L^–1^), S – concentration of glucose (g L^–1^), t_0_ – initial time of log phase development (h), and t – final time of log phase (h).

### Tolerance to Challenges Mimicking Gastrointestinal Transit

To evaluate *S. boulardii* survivability under *in vitro* gastrointestinal conditions, 10 mL aliquots from the polynomial mathematical model validation experiment samples were collected as described above. *In vitro* GI treatment was carried out as reported by Guerra et al. (2018) and Lemos Junior et al. (2019). GI base juice was formulated as follows: 0.11 g L^–1^ calcium chloride, 1.12 g L^–1^ potassium chloride, 2.0 g L^–1^ sodium chloride, and 0.4 g L^–1^ potassium dihydrogen phosphate. This solution was sterilized at 121°C for 15 min. Artificial gastric juice (GJ) was freshly prepared (75 mL) by adding 3.5 g L^–1^ swine mucin and 0.26 g L^–1^ swine pepsin (SigmaAldrich, S. Louis, MO, United States). The pH was adjusted to 2.0 with 1.0 M HCl. Sample aliquots (1 mL) was transferred into GJ and anaerobically incubated at 36°C for 45 min, with gentle shaking. Subsequently, artificial intestinal juice was obtained by adding 3.0 g L^–1^ bile salt, 1.95 g L^–1^ pancreatin, and 0.1 g L^–1^ egg white lysozyme (SigmaAldrich, S. Louis, MO, United States) to the GJ. Sterile distilled water was used to top up 100 mL final volume. The pH was adjusted to 7.0 with 1.0 M sodium bicarbonate solution and anaerobically incubated at 36°C for 180 min, with gentle shaking. 12-wells microtiter plate with WL agar (SigmaAldrich, S. Louis, MO, United States) was used to count viable cells by drop plate technique both after growing in YPD medium and gastric and GI treatment, so that each well was seeded by only a drop from each dilution level – until 10^–6^. Plates were incubated at 30°C for 48 h, under aerobic condition. Colonies were quantified with the aid of colony counter. The results were expressed as weighted (Log_10_ cfu mL^–1^), using at least two successive dilutions level, according to ISO 7218 (2007).

### Statistical Analysis

The results were reported as the mean ± standard deviation (SD) (*n* = 3) and analyzed by a variance test (ANOVA) followed by Fischer’s or Dunnett’s test. Statistica software version 10.0 (StatSoft, Tulsa, United States) was used for the central composite rotatable design analyses. A desirability function was used to obtain the preferred conditions of the dependent variables as a function of cellular concentration in dry weight and viable cells. Action Stat software version 3.1.43.724.694 (ESTATCAMP, Brazil) was used for other statistical analyses. All statistical analyses were performed at 95% confidence.

## Results

### Screening on the Laboratory Scale

**FIGURE 1:**
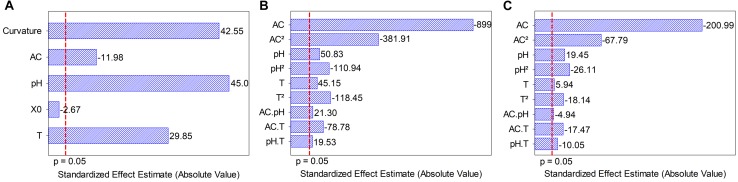
**(A)** Pareto chart of the standardized effects of independent variables on biomass formation. **(B)** Pareto chart of standardized effects from model terms for the independent variables on biomass formation. **(C)** Pareto chart of standardized effects from model terms for the independent variables on *Saccharomyces boulardii* viability. AC, alcohol content (°GL); pH, potential of hydrogen; X_0_, initial *S. boulardii* concentration (cells mL^–1^); T, temperature (°C).

The screening results revealed that alcohol content had a negative effect on dry weight, whereas temperature and pH had a positive effect on dry weight. Initial *S. boulardii* concentration was not significant (Figure 1A). The Plackett and Burman design showed a significant curvature (*p* < 0.05), indicating that the optimal point was among the levels tested. Three independent variables (pH, temperature and alcohol content) were defined as significant for the central composite rotatable design optimization.

The initial *S. boulardii* concentration effect was lower than that of other variables (approximately 4.5, 16.9 and 11.2 times lower than the effects of alcohol content, pH, and temperatura, respectively) and had no significant influence on dry weight. Based on the Plackett and Burman results, temperature (16.8 up to 25.2°C) and pH (4.66 up to 6.34°C) were adjusted to maximize the growth of *Saccharomyces boulardii*, and alcohol content (0.0 up to 14.8°GL) was extended to determine its growth ability in the presence of alcohol to maximize cellular concentration in dry weight and viable cells in central composite rotatable design performance. All coefficients (Figures 1B,C) from the polynomial mathematical models show statistical significance for cellular concentration in dry weight and viable cells, including the interactions among independent variables (*p* ≤ 0.05), determination coefficient (*R*^2^) values (0.9154 and 0.9347) and adjusted *R*^2^ (0.8308 and 0.8693). A p (lack of fit) <0.0001 indicates the adequacy of the models, Eqs (9) and (10).

(9) $$X_{\mathrm{𝑑𝑤}}=-18.3197+0.1809\,\mathrm{𝐴𝐶}-0.0166\,{\mathrm{𝐴𝐶}}^{2}+3.7762\,\mathrm{𝑝𝐻}-0.3745\,{\mathrm{𝑝𝐻}}^{2}+0.6506\,T-0.0160\,T^{2}+0.0107\,\mathrm{𝐴𝐶}\,\mathrm{𝑝𝐻}-0.0079\,\mathrm{𝐴𝐶}\,T+0.0172\,\mathrm{𝑝𝐻}\,T$$

(10) $$X_{\mathrm{𝑣𝑐}}=-16.4677+0.2604\,\mathrm{𝐴𝐶}-0.0138\,{\mathrm{𝐴𝐶}}^{2}+5.6558\,\mathrm{𝑝𝐻}-0.4131\,{\mathrm{𝑝𝐻}}^{2}+0.7807\,T-0.0115\,T^{2}-0.0116\,\mathrm{𝐴𝐶}\,\mathrm{𝑝𝐻}-0.0082\,\mathrm{𝐴𝐶}\,T-0.0415\,\mathrm{𝑝𝐻}\,T$$

**FIGURE 2:**
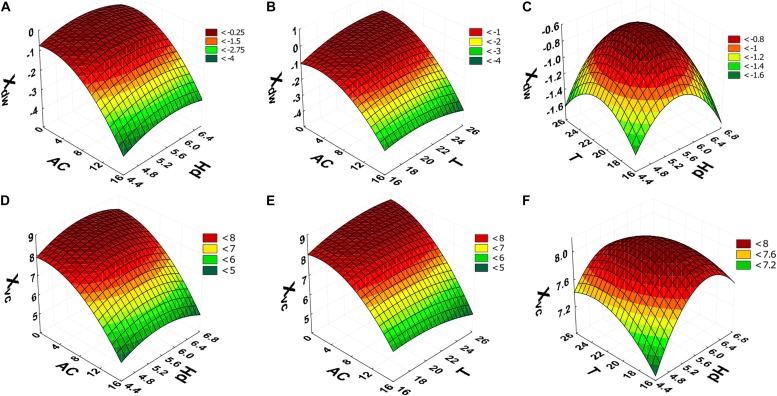
Central composite rotatable design (CCRD 2^3^) response surfaces. X_dw_ dependent variable **(A–C)**; results to X_vc_ **(D–F)**. AC, alcohol content (°GL). pH, potential of hydrogen; T, temperature (°C); Xdw, *S. boulardii* biomass formation (Log_10_ g in dry weight L^–1^); X_vc_, viable *S. boulardii* concentration (Log_10_ viable cells mL^–1^).

**FIGURE 3:**
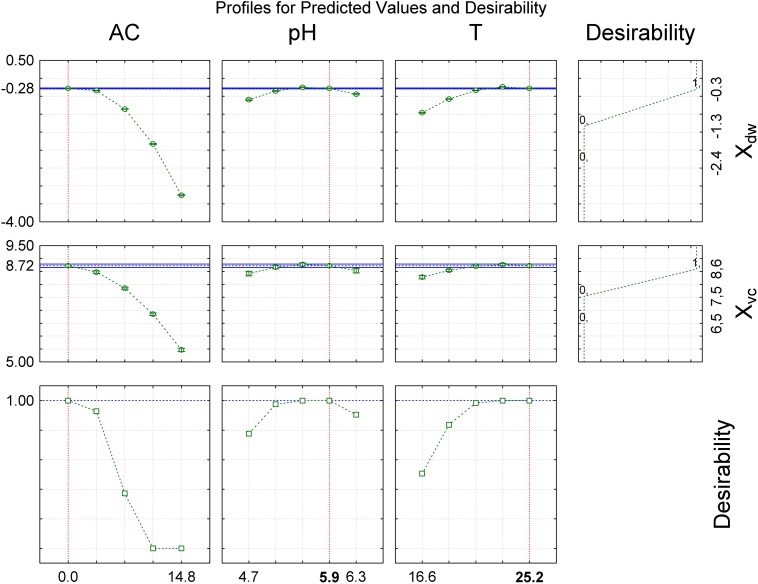
Desirability function to evaluate the influence of independent variables to maximize the growth of *S. boulardii*. AC, alcohol content (°GL); pH, potential of hydrogen; T, temperature (°C); X_dw_, *S. boulardii* biomass formation (Log_10_ g in dry weight L^–1^); X_vc_, viable *S. boulardii* concentration (Log_10_ viable cells mL^–1^).

The surface response graphs (Figures 2A–F) show that the ranges of alcohol content, pH and temperature were adequate to maximize cellular concentration in dry weight and viable cells, indicating that the laboratory-scale experiment was optimized. In accordance with the polynomial mathematical models and maximum value of desirability function (*d*_i_) (0.00 ≤ *d*_i_ ≤ 1.00), cellular concentration in dry weight and viable cells were maximized with alcohol content (0.0°GL), pH (5.92), and temperature (25.2°C) (Figure 3).

### Validation of the PMM in a 5 L Bioreactor

For polynomial mathematical model validation, the stationary phase was considered at the first time point (37.5 h), where dry weight was −0.61 Log_10_ g dry weight L^–1^. The predicted value from polynomial mathematical models was −0.37 Log_10_ g dry weight L^–1^; therefore, a large relative deviation (63.74%) was observed. However, the viable cell concentration values in the laboratory experiment (7.63 Log_10_ viable cells mL^–1^) and the polynomial mathematical model (8.43 Log_10_ viable cells mL^–1^) were closer, with a relative deviation of 9.54%.

**FIGURE 4:**
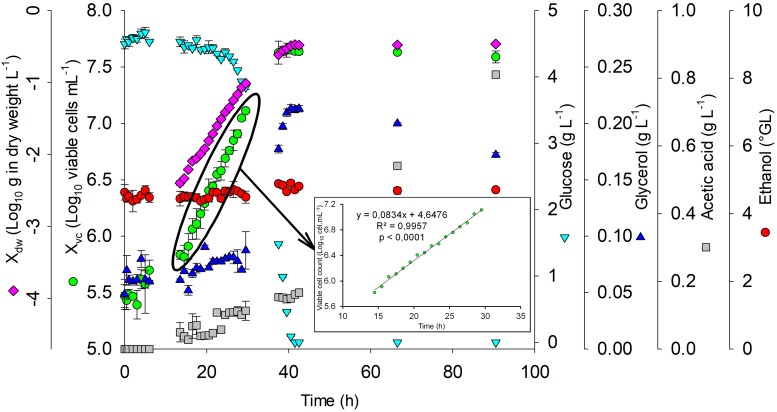
Growth kinetics of *S. boulardii*. Glucose and ethanol consumption and acetic production for confirmation of the mathematical model carried out with an alcohol content = 5°GL, pH = 5.5, and temperature = 24°C. X_dw_, *S. boulardii* biomass formation (Log_10_ g in dry weight L^–1^); X_vc_, viable *S. boulardii* concentration (Log_10_ viable cells mL^–1^); g L^–1^, glucose; g L^–1^, glycerol; g L^–1^, acetic acid; °GL, ethanol.

The adaptation phase (lag) for polynomial mathematical model validation was up to 3 h after inoculating *S. boulardii*. The growth acceleration phase was from 3 to 14.5 h, followed by the exponential growth phase up to 35.76 h (Figure 4), in accordance with linear regression (*R*^2^ = 0.9957 e *p* < 0.0001). The values of t_g_ (3.50 h) and μ_X_ (0.20 h^–1^) were calculated from the log phase.

During the exponential growth phase, acetic acid production (0.15 g L^–1^), glycerol production (0.21 g L^–1^), and an increase in *S. boulardii* concentration (5.47 to 7.63 Log_10_ cells mL^–1^) occurred, in addition to reductions in glucose (5.35 up to 4.53 g L^–1^), dissolved oxygen (6.63 up to 0.16 ppm), and pH (5.35 up to 4.53). At the end of the exponential growth phase, all the glucose was consumed, and the acetic acid production had increased to 0.81 g L^–1^.

**FIGURE 5:**
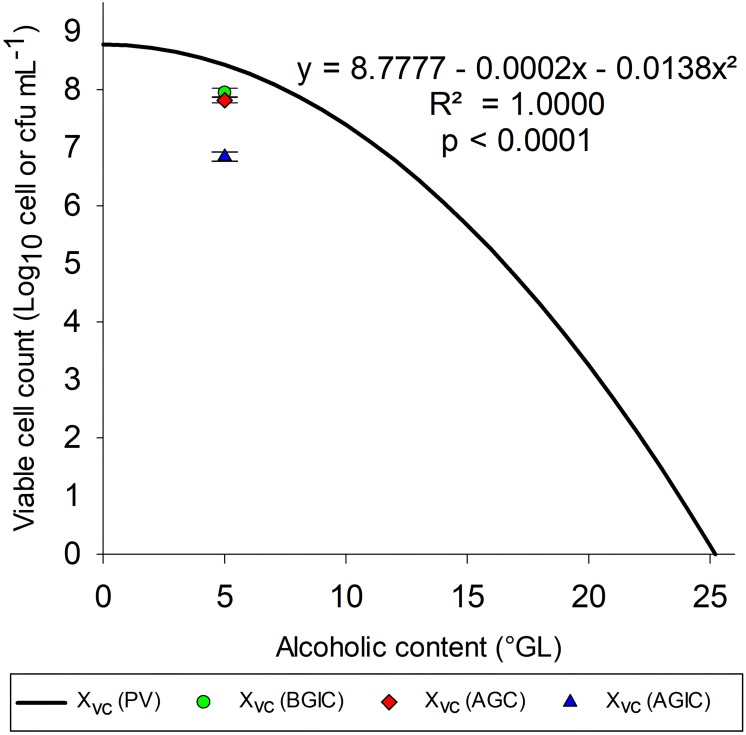
Tolerance of *S. boulardii* to medium containing ethanol. X_vc_ (PV) = viable cell concentration (Log_10_ viable cells mL^–1^) values predicted by the mathematical model with pH and temperature set to 5.5 and 24°C, respectively; X_vc_ (BGIC), viable cell concentration before gastrointestinal conditions (Log_10_ cfu mL^–1^); X_vc_ (AGC), concentration of viable cells after gastric conditions (Log_10_ cfu mL^–1^); X_vc_ (AGIC), concentration of viable cells after gastrointestinal conditions (Log_10_ cfu mL^–1^).

Figure 5 shows the polynomial mathematical model with fixed pH (5.5) and T (24°C) to evaluate the growth of *S. boulardii* at several alcohol contents. After polynomial mathematical model validation with fixed pH and temperature, it was possible to verify that with alcohol content 11.3°GL, the polynomial mathematical model indicates an viable cells concentration of 7.0 Log_10_ cells mL^–1^.

## Discussion

*Saccharomyces boulardii* is more tolerant of acidic pH and temperature variation than other *S. cerevisiae* strains and can survive at pH values as low as 2.0 (Moradi et al., 2018). Often, yeasts are less susceptible to ethanol than other microorganisms, especially bacterial genera. Otherwise, alcohol may modify the cell membrane permeability and negatively affect nutrient transport (Henderson and Block, 2014).

The variation between results from the polynomial mathematical model (10 mL screw-cap tubes) and the confirmation test (5 L bioreactor) can be explained by differences such as the volume (10 mL against 5 Land geometric design, which is the constant relation between the corresponding linear dimensions in the two scales (diameter and height). Inconsistent environmental conditions sometimes lead to different and unsatisfactory results at the laboratory or pilot scale due to physiological changes in the microorganism activity (Pereira et al., 2008). When the experiment was carried out in a screw-cap tube (10 mL), the biomass formation was higher than that produced in the bioreactor, but the cell number was not. Therefore, the geometric dimensions seem to have a stronger effect on the cell size than on the number of cells. According to Costa (2017), environmental stress conditions, such as alcoholic stress and oxidative stress, lead to metabolic modification aimed at cell protection. Oxidative stress is the response to damage caused by excess reactive oxygen species (superoxide anion, hydrogen peroxide, and hydroxyl radical) or by changes in the cellular redox status (Smart, 2008), and alcoholic stress is the response to the presence of ethanol in the medium, which at low concentrations acts as a growth inhibitor in yeast, inhibiting cell division and decreasing cell volume and growth rate, and at high concentrations reduces vitality and increases cell death (Birch and Walker, 2000). Oxidative stress affects proteins, lipids, and deoxyribonucleic acid (DNA) in different cell structures, and alcoholic stress mainly affects cell membranes, proteins and the endoplasmic reticulum, leading to the expression of genes for response to environmental stress, especially heat shock proteins (Costa, 2017). In the polynomial mathematical model validation experiment, the addition of ethanol and correction of the pH of the culture medium were performed in the bioreactor under constant stirring (160 rpm), causing oxygenation in the culture medium, which together with the difference in the geometric design of the reactor and the presence of ethanol may have increased cell stress, causing the smaller size of *S. boulardii* cells in comparison to that in the screening tests.

Trigueros et al. (2016) developed assays for the modeling and optimization of culture medium using hydrolyzed cheese whey permeate as a substrate for fermentation with *S. boulardii*. The models showed that the optimal pH for microorganism growth was between 4.5 and 5.5 with specific growth rate (μ_X_) equal to 0.17 h^–1^. The experiment was carried out in medium without ethanol incubated at 30°C. Thus, the value obtained by calculating rate of substrate utilization (μ_S_) indicates that in the exponential phase, 3.13 × 10^–12^ g glucose was consumed per cell of *S. boulardii* per hour. Similarly, the value obtained for conversion factor of substrate into biomass (Y_X/S_) indicates that for *S. boulardii*, the growth of 1.42 × 10^10^ cells occurred per g of glucose consumption.

Muller et al. (2007) compared *S. boulardii* growth in air lift and shaker bioreactors, obtaining values of specific growth rate between 0.350 and 0.382 h^–1^ for with an initial pH of 6.0 and incubation at 30°C in the absence of ethanol in the culture medium. The values reported by Muller et al. (2007) demonstrate consistency with the values obtained in this work, especially in a culture medium containing ethanol.

McFarland (2017) prescribed a minimum (9.0 Log_10_ cell) dose of *S. boulardii* to confer health benefits on humans. Thus, probiotic alcoholic beverage benefits may be obtained from the intake of approximately 100 mL of the drink, but this value depends on its alcohol content. In this work, *S. boulardii* survivability showed a slight reduction of 0.13 Log_10_ cfu mL^–1^ after gastric conditions and 0.97 Log_10_ cfu mL^–1^ after intestinal conditions (total reduction of 1.10 Log_10_ cfu mL^–1^), with a survivability of 6.85 Log_10_ cfu mL^–1^ after gastrointestinal conditions in culture medium with 5.0°GL.

Enough viable probiotics are necessary to play a beneficial role on the host (Joint FAO/WHO Working Group, 2002). Gastrointestinal resistance is also an important property to probiotics when gut microbiome balance is the target; however, only alive microorganisms can confer additional benefits. Beer is so stressful matrix to the microorganisms, once alcohol and acids produced during the fermentation can damage cells over storage period. Thus, GI transit is an overlapping stress factor to the yeast after brewing. Probiotic alcoholic beverage benefits may be obtained from the intake of approximately 100 mL of the drink, but this value depends on its alcohol content. In this work, *S. boulardii* survivability showed a slight reduction of 0.13 and 0.97 Log_10_ cfu mL^–1^ after gastric conditions and intestinal conditions, respectively, with a survivability of 6.85 Log_10_ cfu mL^–1^ after gastrointestinal transit in culture medium with 5.0°GL.

According to Stackelberg et al. (2014), moderate alcohol consumption may reduce the risk of abdominal aortic aneurysm in both men and women. Therefore, moderate alcohol consumption is not harmful, and non-distilled alcoholic beverages may have protective properties against the development of abdominal aortic aneurysm. The effect of higher doses of alcohol on the risk of disease remains unknown. Moderate consumption standard: one standard dose (12 g ethanol) was calculated as 150 mL of wine, 80 mL of strong wine, 660 mL of Class I beer (<2.25°GL), 500 mL of Class II beer (2.25–3.5°GL), 330 mL of Class III beer (≥3.5°GL), or 40 mL of liquor. Markus et al. (2015) found that light to moderate alcohol consumption was associated with a lower risk of developing aortic valve sclerosis both in men and women. These results are in accordance with the objective of this work, which was to optimize the growth of *S. boulardii*, allowing the development of probiotic alcoholic beverage to obtain additional benefits. According to the results obtained using the polynomial mathematical model, it is possible to produce a probiotic wine with alcohol content equal 11.9°GL, obtaining viable cell concentration 6.82 Log_10_ viable cells mL^–1^. Following the criteria adopted by Stackelberg et al. (2014) of moderate wine consumption, 150 mL allows the ingestion of 9.00 Log_10_ viable cells, which corresponds to the minimum value indicated by McFarland (2017). Similarly, for the results obtained in the polynomial mathematical model validation test (7.63 Log_10_ viable cell mL^–1^) with alcohol content equal 5°GL, corresponding to a Class III beer, it would be necessary to ingest only 23.44 mL, well below 330 mL (Stackelberg et al., 2014), to meet the minimum concentration indicated by McFarland (2017).

The minimum sensory limit for acetic acid perception is 0.07 g L^–1^ (Zhang et al., 2012). Therefore, stopping the fermentation before the stationary phase is extremely important to ensure that acetic acid production is lower than the perception sensory limit. Often, the acetic acid concentration in different styles of beers ranges from 0.06 to 0.15 g L^–1^. At the beginning of the stationary phase, the glycerol concentration was 0.18 g L^–1^. According to Zhao et al. (2015), glycerol is significantly related to the density and viscosity of beer, and its sensory perception level is 10 g L^–1^.

*Saccharomyces boulardii* may have good potential for probiotic alcoholic beverage development. Slight microbial susceptibility to gastrointestinal conditions was observed, and alcohol exhibited no severe stress on *S. boulardii* survivability. The results provide new insight into the management of probiotic beverages and suggest new prospects for a more integrated strategy for increasing beverage quality.

## Data Availability

All datasets generated for this study are included in the manuscript and/or the supplementary files.

## Author Contributions

BP participated in the planning, conducted the experiments, and wrote and revised the manuscript. DC assisted in the statistical analysis and revised the manuscript. WL and AG assisted in the microbiological analyses and reviewed the manuscript. MCor performed the chromatographic analyses and revised the manuscript. KP and MCoe planned and coordinated the experiments, and revised the manuscript.

## Conflict of Interest Statement

The authors declare that the research was conducted in the absence of any commercial or financial relationships that could be construed as a potential conflict of interest.
